# Broadband Superabsorber Operating at 1500 °C Using Dielectric Bilayers

**DOI:** 10.1021/acsaom.3c00229

**Published:** 2023-09-08

**Authors:** Tao Gong, Margaret A. Duncan, Micah Karahadian, Marina S. Leite, Jeremy N. Munday

**Affiliations:** † Department of Materials Science and Engineering, University of California, Davis, California 95616, United States; ‡ Department of Electrical and Computer Engineering, University of California, Davis, California 95616, United States

**Keywords:** extreme environments, photonics, FTIR, superabsorption, high temperature, thermophotovoltaic

## Abstract

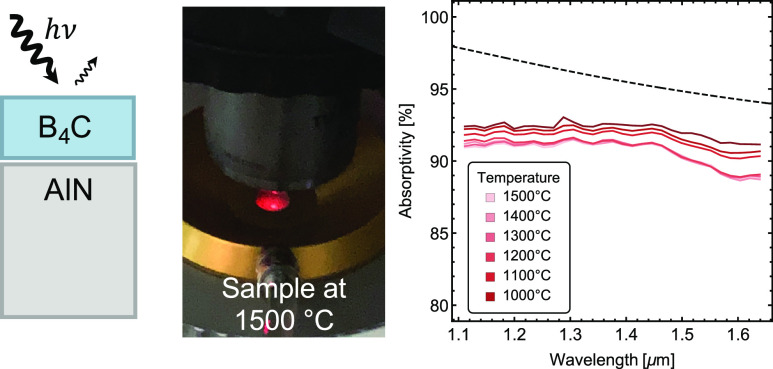

Many technological applications in photonics require devices to function reliably under extreme conditions, including high temperatures. To this end, materials and structures with thermally stable optical properties are indispensable. State-of-the-art thermal photonic devices based on nanostructures suffer from severe surface diffusion-induced degradation, and the operational temperatures are often restricted. Here, we report on a thermo-optically stable superabsorber composed of bilayer refractory dielectric materials. The device features an average absorptivity ∼95% over >500 nm bandwidth in the near-infrared regime, with minimal temperature dependence up to 1500 °C. Our results demonstrate an alternative pathway to achieve high-temperature thermo-optically stable photonic devices.

## Introduction

While there have been tremendous advances in photonic technologies over the last decade, their operation under extreme conditions and environments, including high temperatures, is still in its infancy. For instance, as the size of photonic devices shrinks with increasing chip-scale integration and compactness, plasmonic or photonic resonances in these devices are often accompanied by substantial local heating at the “hot spots” due to strong electromagnetic field confinement.^1,2^ In addition, a wealth of thermal photonic applications by their nature call for reliable high-temperature performances, such as thermophotovoltaics (TPVs), radiative cooling, photothermal tumor ablation, heat-assisted magnetic recording, and optical devices with high input intensities.^3−8^ These applications generally require the device architecture and the constituent materials to possess thermally stable optical, mechanical, and chemical properties. In actuality, state-of-the-art thermal photonic devices (e.g., thermal emitters in TPV systems) are typically constructed using refractory metals (e.g., W, Ta, and Mo) and dielectrics (e.g., certain nitrides, carbides, and oxides) with high melting points, which are highly resistant to degradation at high temperatures.^5,9−11^

From the perspective of device structures, two primary categories have been explored extensively for thermal photonic applications: bulk refractory materials (for broadband graybody emitters such as SiC, graphite, and W)^12−18^ and nanostructured materials for selective emitters.^19−28^ The former usually exhibits broadband emissivity (or equivalently broadband absorptivity, according to Kirchhoff’s law)^29^ over the wavelength range of interest for most TPVs, which helps improve the output power density for the cell due to the large radiated power from the emitter. The latter often features a resonant emissivity/absorptivity spectra and results in a better power conversion efficiency, provided the emission spectrum is tailored to match the band gap of the PV cell such that out-of-band photon emission is considerably suppressed.^30,31^ However, they both have respective technological constraints: the broadband emitter is structurally simple, yet possible materials are limited and are not necessarily well-suited for device integration. The selective emitters often entail time-consuming nanopatterning processes owing to their structural complexity (e.g., in photonic crystals, metamaterials, nanoantenna, and gratings). Moreover, the thermal stability of nanostructures is usually worse than their bulk counterparts due to accelerated surface diffusion at the curvature edges, and henceforth, the operating temperature is often restricted (typically below 1000 °C).^32^

In this work, we report a near-infrared (NIR) superabsorber consisting of bilayer refractory dielectric materials: B_4_C/AlN. The device features a broadband absorption with an average absorptivity of ∼95% over a 500 nm wavelength span in the NIR wavelength regime (1.10–1.65 μm), which we term superabsorption. In addition, this dielectric bilayer is stable during an 8 h thermal treatment in a low-oxygen environment, with negligible change of its optical characteristics for temperatures up to 1500 °C, demonstrated by in situ thermal emission spectra measurements. The experimental results are corroborated by optical simulations. Our work creates new opportunities for realizing thermal photonic devices using alternative refractory materials. While most reports thus far have focused on postmortem analysis of the sample after heating treatments, we present a unique setup for in situ optical measurements at extreme temperature conditions. This setup can be modified to probe the effects of distinct surroundings (e.g., inert environment, vacuum, and oxygen-rich ambient) on the optical behavior of materials during heating and cooling processes. Overall, these measurements are critical for selecting materials for photonic devices that will operate at high temperatures and/or be exposed to these conditions.

## Results and Discussion

The superabsorber considered here is a bilayer structure: B_4_C thin film coated on an AlN substrate. Both dielectric materials have a melting temperature >2000 °C with outstanding thermal stability. The AlN substrate is 0.5 mm thick (single-side polished), and a 135 nm thick B_4_C layer is sputtered on top conformally covering the substrate. Besides the material stability, structural robustness of such a bilayer device has been predicted because of the minimal thermal expansion mismatch of the two materials and the mild interlayer diffusion at their interface.^33^

**Figure 1:**
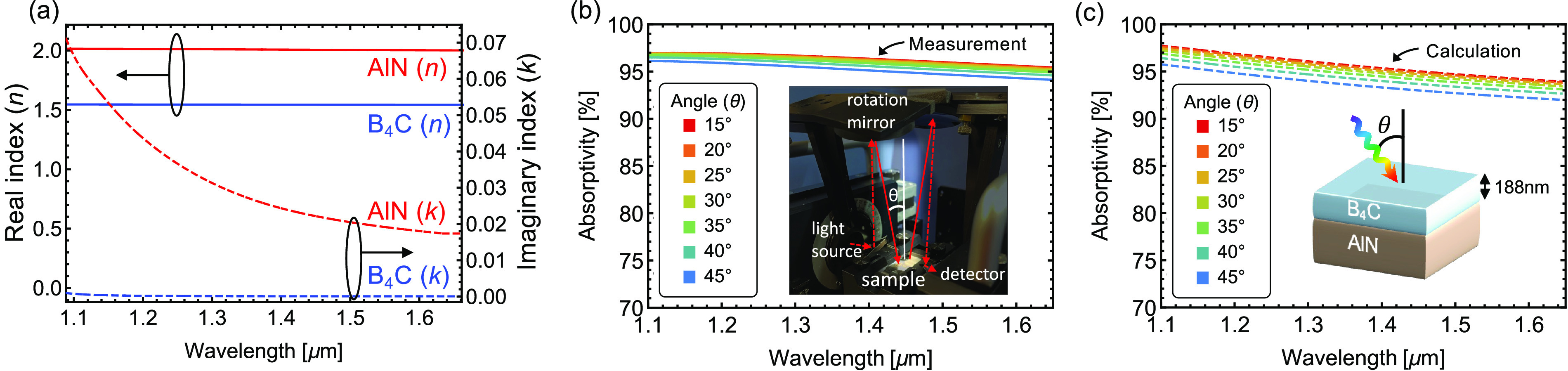
(a) Refractive indices (solid lines for the real part and dashed lines for the imaginary part) for AlN (red) and B_4_C (blue). (b) Measured and (c) calculated absorptivity of the device at different illumination angles. Inset of (b) shows photograph of optical path in the FTIR setup. Inset of (c) displays schematic of superabsorber structure: the B_4_C thin film (135 nm thick) is sputtered onto the AlN substrate.

The room-temperature refractive indices (*n* + i*k*) of the layers are determined by spectroscopic ellipsometry. As shown in Figure 1a, the real part of the refractive index *n* of B_4_C (∼1.54) falls between that of air (∼1) and AlN (∼2.01), which tends to suppress reflection at the top surface. This anti-reflection effect in combination with the small but nontrivial loss of AlN (imaginary index *k* ∼ 0.02) can theoretically result in a large absorptivity in the structure (with calculated transmission ≪0.01%), as will be confirmed in optical measurements discussed below. The absorptivity of the device at varying incident angles is measured using Fourier-transform infrared spectroscopy (FTIR) for unpolarized light (see inset of Figure 1b for a photograph showing the optical path of the FTIR). As shown in Figure 1b, the measured absorptivity is consistently over 95% across the NIR wavelengths with negligible dependence on the incidence angle. Our calculations using the transfer-matrix-method (TMM) are in excellent agreement with the measurements (Figure 1c), confirming the angular insensitivity of such bi-layer optical devices.

**Figure 2:**
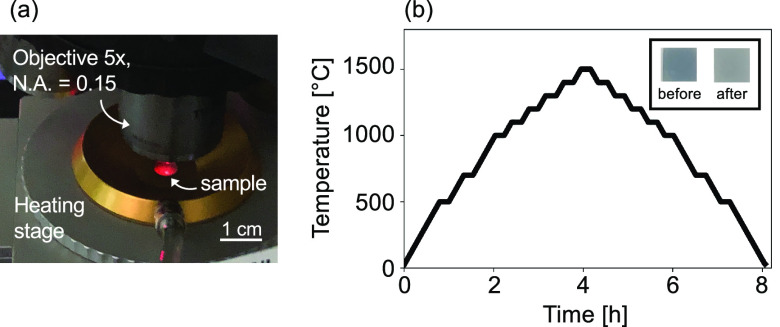
(a) Photograph of the optical setup for high-temperature in situ optical measurements. (b) Temperature profile used during high-temperature experiments. Inset: photograph of the B_4_C/AlN optical device before and after the treatment.

**Figure 3:**
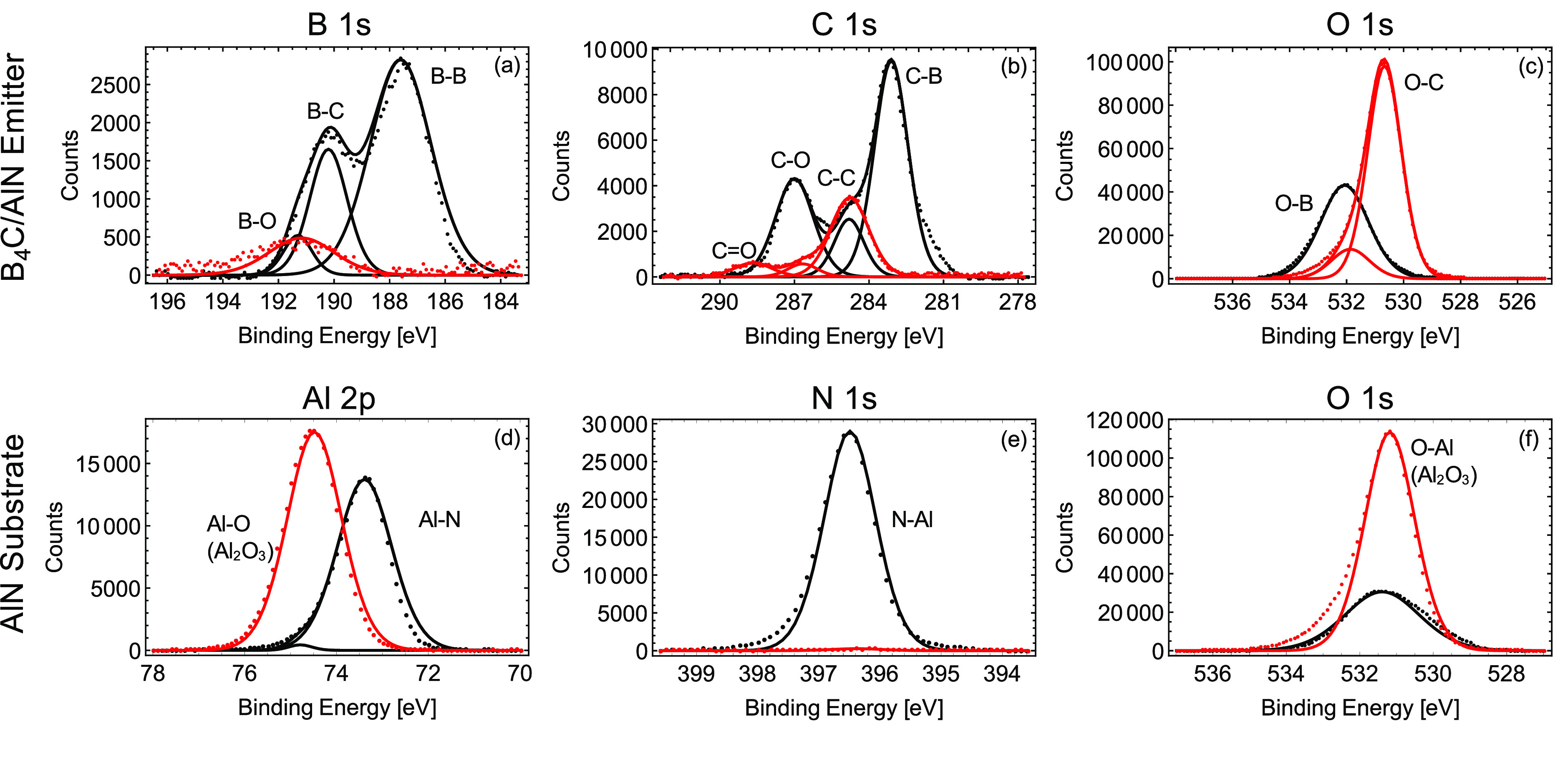
XPS measurements for (a–c) 135 nm B_4_C/AlN and (d–f) pure AlN substrate, before (black) and after (red) high-temperature treatment. Experimental data and peak fits are represented by dotted curves and solid lines, respectively.

After performing room-temperature optical measurement, we implemented a controlled heating treatment to determine the high-temperature optical behavior of the structure in an inert (argon) environment. Figure 2 shows the heating stage as well as the temperature profile for the heating treatment. In situ emission and reflectivity measurements are performed through a sapphire chamber window, allowing us to analyze the high-temperature performance of the samples in real time. The inset of Figure 2b shows real-color photographs of the sample before and after high-temperature treatment. Slight changes in the coloration are noticeable, as a direct result of a chemical change at the surface of the sample, as discussed in Figure 3.

The color change observed on the sample upon heating treatment (see inset in Figure 2b) results from a modification of the B_4_C surface. We determine these chemical changes by comparing pristine and temperature-cycled samples using X-ray photoelectron spectroscopy (XPS). Figure 3a–f displays the changes in the chemical composition for the surfaces of 135 nm B_4_C/AlN and the pure AlN substrate, respectively, before (black) and after (red) identical heating/cooling. Here, measured data are shown with dots, and fitted curves and their constituent peaks are presented as solid lines. Before the high-temperature experiments, both samples show evidence of an ultra-thin native oxide layer, confirmed by the presence of expected oxide peaks in Figure 3. However, due to the presence of characteristic peaks for B_4_C (Figure 3a,b) and AlN (Figure 3d,e), the surface oxide layer for both samples must be less than 10 nm thick prior to high-temperature treatment, given the limited surface penetration depth of XPS.^34^ Both samples undergo further surface oxidation as a result of high-temperature operation, with a final top oxide layer of at least 10 nm, confirmed by the disappearance of the C–B peaks for the B_4_C-coated sample (Figure 3c,d)^35,36^ and by the shift of the Al peak for the pure AlN substrate (Figure 3f).^37^ These data reveal the high preference of the samples for oxidation. Even in a low O_2_ environment (<0.1% oxygen pressure), they preferentially oxidize to form a thin oxide layer at the surface.

**Figure 4:**
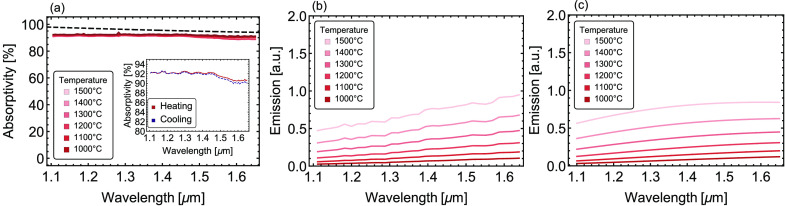
(a) Measured (solid lines) and calculated (dashed line) absorptivity of the B_4_C/AlN optical device at varying temperatures, as color coded. Inset: the absorptivity at 1000 °C during heating and cooling, respectively. (b) Measured and (c) calculated emission spectra of the sample at varying temperatures.

Despite the small surface chemical changes, the optical absorptivity of the B_4_C/AlN device exhibits impressive stability at high temperatures. Figure 4a shows the absorptivity (*A*) derived from the measured reflectivity (*R*) at varying temperatures from 1000 to 1500 °C in the heating phase (*A* = 1 – *R* because the sample is opaque). The average absorptivity in the wavelength range 1.1–1.65 μm is >90% for all temperatures measured, which results in an excellent broadband superabsorber. Additionally, despite a slight decrease of the absorptivity with increased temperature, the variation of absorptivity within the explored range is small (<5%), which indicates the remarkable thermal stability despite the surface changes during the thermal treatment. Furthermore, the absorptivity is also measured at the same temperatures (1000–1500 °C) during the cooling process. Very minimal change is observed at the same temperature during heating and cooling. For example, the inset of Figure 4 a shows the respective absorptivity profiles at 1000 °C in the heating and cooling phase overlap. All above results also indicate that while the surface of the device oxidizes after the thermal treatment (as shown through the changing peak heights and locations in Figure 3), the optical properties in the NIR regime are not significantly affected. Concomitantly, the structure can also act as an excellent high-temperature graybody emitter. According to Kirchhoff’s law, the emissivity of a device is equal to the absorptivity; thus, the broadband absorptivity of our device should yield a high-temperature graybody emitter. Figure 4b displays the measured in situ emission spectra at temperatures from 1000 to 1500 °C, which agree quite well with theoretical calculations of emission shown in Figure 4c.

## Conclusions

In summary, we demonstrate excellent thermo-optical stability of a B_4_C/AlN bilayer when acting as an NIR superabsorber or super-emitter, based on a scalable design. The device features broadband, angle-insensitive super-absorption in the NIR wavelength range, with an average absorptivity of greater than 90%. We present both experimental and calculated results verifying the optical stability of the device at high temperatures, including in situ absorptivity and emission at temperatures up to 1500 °C. The measured thermal emission spectra at high temperatures are in good agreement with our theoretical predictions. Though we note the presence of surface oxidation during high-temperature treatment, the sample still presents broadband absorption at high temperatures. Our results show great promise for achieving optically stable photonic devices under high-temperature environments using alternative refractory materials. The pressing need for materials under extreme temperature conditions is exposing the need for detailed in situ characterization of the optical behavior of materials, which is frequently limited to postmortem analyses, after samples’ exposure to heating treatments. Here, we implement in situ, high-temperature optical measurements that could be expanded to different photonic systems, ranging from optical emitters for TPVs (in air or vacuum conditions) to barrier coatings for aerospace applications, where identifying the effects of distinct surroundings on materials’ absorptivity during heating and cooling is critical.

## Experimental Methods

### Sample Fabrication

A 135 nm thick B_4_C layer was sputtered on top of a 0.5 mm thick, single-side polished AlN dielectric substrate (MTI Corporation) using a Lesker LabLine RF sputter system and a B_4_C sputter target, conformally covering the substrate. Sample uniformity was confirmed using spectroscopic ellipsometry at different points across the sample, as well as optical microscopy.

### Room-Temperature Optical Characterization

Room-temperature optical properties were taken using a J. A. Woollam M-2000 spectroscopic ellipsometer. Refractive indices were determined by fitting the measured ellipsometric parameters Ψ and Δ. General oscillator models were used to fit both the AlN substrate and the B_4_C coating. The room-temperature angle-dependent absorptivity of the samples was determined using a Bruker Invenio FTIR system. The absorptivity was calculated using measured reflectivity, as *A* = 1 – *R* (because transmission is approximately 0 through these samples).

### Room-Temperature Chemical Characterization

XPS measurements were taken with a Kratos SUPRA Axis XPS, using a monochromated Al Kα source (1486.6 eV). During all measurements, the chamber’s base pressure was 2.1 × 10^–8^ Torr, with a 450 × 900 μm scan size and an emission current of 7 mA.

### High-Temperature Optical Characterization

High-temperature thermal treatment and the in situ optical measurement during the treatment were performed using a Linkam heating stage (TS1500) in conjunction with a Nikon microscope. The device was placed inside the ceramic sample cup on the heating stage (with programmed temperature control up to 1500 °C) so that it could be heated from underneath as well as from the sides to ensure uniform heating. The device surface was brought to the focal point of the objective attached to the microscope. Light reflected off and radiated from the device was collected through the objective (5× magnification, N.A. = 0.15) and subsequently fed into an optical fiber, which connected to an NIR spectrometer (Ocean Insight Flame-NIR+). During the thermal treatment, the temperature was increased to 1500 °C at the rate of 10 °C/min in the heating phase and then decreased to the room temperature at the same rate in the cooling phase. Above 500 °C in both the heating and cooling phases, the sample is held at several temperatures to allow the sample to thermalize before continuing heating/cooling. The spectral data were recorded during the heating and cooling processes while holding at specific temperatures to ensure stable spectral data. The measured emission and absorption data were averaged every three points to minimize noise. The entire thermal treatment lasted for just over 8 h. Argon was supplied to the sample chamber on the heating stage to ensure a low-oxygen atmosphere (<0.1% oxygen pressure) throughout the experiment.
